# Antimicrobial, Antibiofilm, and Antioxidant Activity of Functional Poly(Butylene Succinate) Films Modified with Curcumin and Carvacrol

**DOI:** 10.3390/ma14247882

**Published:** 2021-12-20

**Authors:** Łukasz Łopusiewicz, Szymon Macieja, Artur Bartkowiak, Mirosława El Fray

**Affiliations:** 1 Center of Bioimmobilisation and Innovative Packaging Materials, Faculty of Food Sciences and Fisheries, West Pomeranian University of Technology Szczecin, Janickiego 35, 71-270 Szczecin, Poland; szmacieja@gmail.com (S.M.); Artur-Bartkowiak@zut.edu.pl (A.B.); 2 Department of Polymer and Biomaterials Science, Faculty of Chemical Technology and Engineering, West Pomeranian University of Technology in Szczecin, Al. Piastów 45, 71-311 Szczecin, Poland; mirfray@zut.edu.pl

**Keywords:** bioactive materials, poly(butylene succinate), curcumin, carvacrol, antibacterials, antibiofilm activity, antioxidants

## Abstract

The use of food industry waste as bioactive compounds in the modification of biodegradable films as food packaging remains a major challenge. This study describes the preparation and bioactivity characterization of poly(butylene succinate) (PBS)-based films with the addition of the bioactive compounds curcumin (CUR) and carvacrol (CAR). Films based on PBS modified with curcumin and carvacrol at different concentration variations (0%/0.1%/1%) were prepared by solvent casting method. The antioxidant, antimicrobial, and antibiofilm properties were investigated against bacteria (*Escherichia coli*, *Staphylococcus aureus*) and fungi (*Candida albicans*). As a result of the modification, the films exhibited free radicals scavenging (DPPH up to 91.47% and ABTS up to 99.21%), as well as antimicrobial (6 log, 4 log, and 2 log reductions for *E. coli*, *S. aureus*, and *C. albicans*, respectively, for samples modified with 1% CUR and 1% CAR) activity. Moreover, antibiofilm activity of modified materials was observed (8.22–87.91% reduction of biofilm, depending on bioactive compounds concentration). PBS films modified with curcumin and carvacrol with observed bifunctional properties have many potential applications as active packaging.

## 1. Introduction

The massive production of plastics and the problems with their management after use contribute to environmental pollution on many levels [1,2]. A promising alternative to traditional plastic-based materials may be biodegradable packaging. Groups such as polymers derived from biomass (polysaccharides, proteins, and lipids), polymers from biobased monomers, and polymers produced by microorganisms can be distinguished as potential alternatives to plastics [3,4].

A biodegradable aliphatic polyester, poly(butylene succinate) (PBS), is of increasing interest due to its interesting physical and biological properties. This is because its physicochemical and thermal properties are similar to the polyolefins (polyethylene and polypropylene) traditionally used in the industry. PBS is a derivative of succinic acid (SA), which can be obtained from renewable resources or by microbial fermentation [5]. Furthermore, PBS is able to biodegrade under aerobic conditions, making it a more environmentally friendly, promising material for packaging applications, which is in accordance with the European Green Deal [6,7]. However, some of its disadvantages, such as slow crystallization rate, low melt viscosity, and softness, have limited its processability and applications, especially in injection molding [8,9]. In recent years, many attempts have focused on developing PBS-based blends and composites modified with natural compounds and/or inorganic additives to obtain improved properties and added value in terms of bioactivity (such as antimicrobial and antioxidant) [10].

The increasing bacterial resistance to antibiotics and the occurrence of foodborne infections, as well as the emergence of new mutations of microorganisms, pose a global threat to consumer health [11]. Moreover, oxidative degradation is the leading cause of food spoilage (after microbial growth), and the inclusion of natural antioxidants in food packaging is a way to improve the stability of oxidation-sensitive foods [12,13]. Therefore, developing new active packaging materials that can ensure the microbiological safety of food for consumers and extend the shelf life of products has become a challenge [11,14,15]. Such packaging systems typically use active ingredients derived from natural sources that exhibit antioxidant and/or antimicrobial activity [13,15,16]

Carvacrol (2-methyl-5-(1-methylethyl)-phenol) is a monoterpene that, along with thymol, is contained in the essential oils of plants from the genus *Origanum* [17,18]. The outstanding antimicrobial properties of carvacrol are repeatedly mentioned in the literature and are most likely related to the interaction between the hydroxyl group of carvacrol and lipids in the bacterial cytoplasmic membrane, which leads to its destabilization, increased fluidity, and permeability to ions. In fungi, carvacrol is also likely to disrupt membrane integrity and block ergosterol biosynthesis [18]. This antimicrobial activity has been demonstrated for films from various polymeric matrices, such as PLA [19,20], starch [15], polypropylene [21], carboxymethyl cellulose [22], or fruit/vegetable puree/pectin films [23].

Curcumin ((1*E*,6*E*)-1,7-Bis(4-hydroxy-3-methoxyphenyl)-1,6-heptadiene-3,5-dione) is a natural hydrophobic phenolic component of turmeric (*Curcuma longa* L.), and its most common uses are as a spice and in yellow food coloring (E100) [24,25,26,27,28,29]. Curcumin has recently attracted much attention and has numerous applications due to its broad spectrum of biological functions, including its antimicrobial activity and excellent antioxidant potential [30,31]. Curcumin is also considered non-toxic to humans and is safe even in very high doses [29]. However, low water solubility limits the direct biomedical and packaging applications of curcumin, which has already been reported as a bioactive additive for the production of functional polymer films based on low-density polyethylene [32], poly(lactic acid) [19,29,33], cellulose [34], carboxymethyl cellulose [22], carrageenan [35], cellulose acetate [36], collagen [37], PBAT [28,38,39], pectin [40], and gelatin [27,41,42].

As demonstrated in previous studies, functional PBS-based films with value-added antimicrobial, antioxidant, and radicals scavenging activities can be developed by adding a bioactive compound (quercetin) to a polymer matrix [43]. Moreover, as shown in other studies, bioactive compounds possessing multifunctionality (antimicrobial and antioxidant activity) might show synergistic activity when incorporated into packaging materials [22]. With their potential as functional additives, curcumin and carvacrol are promising agents in PBS-based packaging systems, and some synergies can be expected when these compounds are used together. For instance, carvacrol and montmorillonite showed a synergistic antimicrobial effect in biodegradable, starch-based films [15]. The use of synergistic combinations allows the achievement of a desired antimicrobial and antioxidant performance at lower concentrations of the active components [13]. Moreover, carvacrol and curcumin are designated as generally recognized as safe (GRAS) by the United States Food and Drug Administration (FDA) for use as food additives; thus, their use as additives in bioactive biodegradable films appears to be an outstanding alternative for reducing food loss and waste and improving food security [13,43].

To date, there are no studies on PBS films modified with carvacrol (CAR) or curcumin (CUR). The first purpose of the study was to obtain PBS-based film modified with carvacrol and/or curcumin that could be active against microorganisms and prevent the formation of biofilm on polymeric surfaces. In addition, the synergistic effect of the chosen bioactive compounds was examined. Another objective of this study was to determine the antioxidant potential of the tested materials.

## 2. Materials and Methods

### 2.1. Materials

Poly(butylene succinate) (PBS) (FZ91PM BioPBS™) was procured from Mitsubishi Chemical (Tokyo, Japan). Carvacrol (natural, originated from thyme essential oil, 99%, food grade), sodium chloride, disodium phosphate, monosodium phosphate, 2,2-diphenyl-1-picrylhydrazyl (DPPH), 2,2′-azino-bis(3-ethylbenzothiazoline-6-sulfonic acid) (ABTS), potassium persulphate, potassium ferricyanide, trichloroacetic acid, ferric chloride, iron sulphate, and tris(hydroxymethyl)aminomethane were purchased from Merck Chemical (Saint Louis, MO, USA). Curcumin and crystal violet were purchased from Merck (Darmstad, Germany). Chloroform, ethanol, and methanol were procured from Chempur (Piekary Śląskie, Poland). Agar—agar, Plate Count Agar, Potato Dextrose Agar, and Tryptic Soy Broth were acquired from Merck Chemical (Saint Louis, MO, USA). All chemicals were of analytical grade. *Escherichia coli* ATCC25922, *Staphylococcus aureus* ATCC43300, and *Candida albicans* ATCC10231 were procured from ATCC (American Type Culture Collection, Manassas, VA, USA).

### 2.2. Preparation of PBS-Based Films

PBS and chloroform were placed in glass bottles at a ratio of 8 g of PBS per 100 mL of chloroform. Subsequently, the mixtures were stirred (magnetic stirrer Ika, Staufen im Breisgau, Germany, 200 rpm) until the PBS was completely dissolved. Curcumin and carvacrol were then added to the solutions in amounts suitable to obtain concentrations of 0.1/1.0% (*w*/*w*) by weight (for CUR and CAR separately, as shown in Table 1) of PBS used, and then waiting until completely dissolved. The film-forming solutions were cast on glass Petri dishes (90 mm) and dried at 25 °C for 24 h. The dried films were peeled from the plates and conditioned at 25 °C and 50% relative humidity (RH) in a temperature and humidity clean room prior to any testing.

### 2.3. Determination of Films Antimicrobial Activity

Antimicrobial activity analyses were performed based on ASTM E 2180-01 with modifications described elsewhere [22]. In brief, film sections (2.5 cm × 2.5 cm) were cut and sterilized under UV light. Agar slurries, prepared by combining 0.15 g agar-agar and 0.45 g NaCl in 50 mL of distilled water, were sterilized and, when cooled down, combined with the suspensions of microorganisms to obtain a microbial cells concentration equivalent to 0.5 on the McFarland scale. Subsequently, the mixtures (0.5 mL) were aseptically applied on the surface of the samples, then incubated for 24 h at 30 °C with relative humidity at 90%. Afterwards, the samples were aseptically removed from the Petri dishes, transferred into 10 mL of sterile physiological saline (0.9% NaCl), and thoroughly vortexed. The serial dilutions were prepared and cultures were made on Plate Count Agar (*E. coli* and *S. aureus*) and Potato Dextrose Agar (*C. albicans*) media and incubated at 37 °C for 24 h. Results are expressed as mean values with standard deviations.

### 2.4. Determination of Biofilm Formation on Films

The antibiofilm activity of the samples was assayed following the approach described by Barros et al. [44] with our modification. The film sections (1.5 cm × 1.5 cm) were applied to a 12-well cell culture plate and then each was flooded with 4 mL of Tryptic Soy Broth. A 100 µL suspension of microorganisms with a concentration of 1.5 × 10^8^ CFU/mL was then added to each well. The plates were incubated at 37 °C for 24 h with static conditions. Subsequently, the films were carefully removed from the broth and washed gently with distilled water (to remove planktonic cells) and allowed to dry (at 25 °C for 6 h). The samples were then transferred to 15 mL Falcon tubes and 4 mL of 0.1% crystal violet solution were added. The samples were left static for 20 min in the dark. The crystal violet solution was removed and the stained biofilms were washed with distilled water five times to remove excess unbound dye. The samples were transferred to new 15 mL Falcon tubes and 2 mL of 30% acetic acid solution were added to dissolve the dye. The tubes were shaken at 150 rpm in the dark for 25 min. Then, the absorbance values at 595 nm were measured in 96-well plates using a microplate reader (Synergy LX, BioTek, Winooski, VT, USA) against 30% acetic acid as a blank.

### 2.5. Determination of Reducing Power and Free Radicals Scavenging Activity

The reducing power and DPPH and ABTS radicals scavenging activities analysis were assayed following the methodology detailed elsewhere [45]. The reducing power was determined by adding 1.25 mL of phosphate buffer (0.2 M, pH 6.6) to the film samples (100 mg) and then adding 1.25 mL of 1% potassium ferricyanide solution. The samples were then incubated at 50 °C for 20 min before the addition of 1.25 mL of trichloroacetic acid. The sample tubes were centrifuged at 3000 rpm for 10 min and 1.25 mL of the supernatant was then combined with 1.25 mL of distilled water. To finish, 0.25 mL of 0.1% ferric chloride solution was added to the resulting mixtures and the absorbance was read at 700 nm.

DPPH radicals scavenging activity was assayed by placing 100 mg of film samples in 25 mL of 0.01 mM DPPH methanolic solution and incubating for 30 min without light. Subsequently, the absorbance was measured at 517 nm. The DPPH scavenging rate was calculated from the formula:

(1) $$\%\mathrm{DPPH}\;\mathrm{scavenging}\;\mathrm{activity}=100-\frac{{\mathrm{Abs}}_{\mathrm{sample}}\times 100}{{\mathrm{Abs}}_{\mathrm{control}}}$$

where

Abs_sample_—absorbance of solution after incubation with modified films, and

Abs_control_—absorbance of solution after incubation with neat film.

ABTS radicals scavenging activity was determined by placing 100 mg of film samples in 10 mL of ABTS radicals solution (produced by mixing 7 mM ABTS with 2.45 mM potassium persulfate) and incubating in the dark for 6 min. Then, the absorbance at 734 nm was measured. The ABTS scavenging rate was calculated according to the same equation as the DPPH method.

### 2.6. Statistical Analysis

Statistical analyses were conducted using Statistica version 10 (StatSoft Polska, Kraków, Poland). Differences between means were tested by Fisher’s LSD post hoc test with a significance threshold of *p* < 0.05. All measurements were performed in triplicate.

## 3. Results

### 3.1. Antimicrobial and Antibiofilm Activity

**Figure 1:**
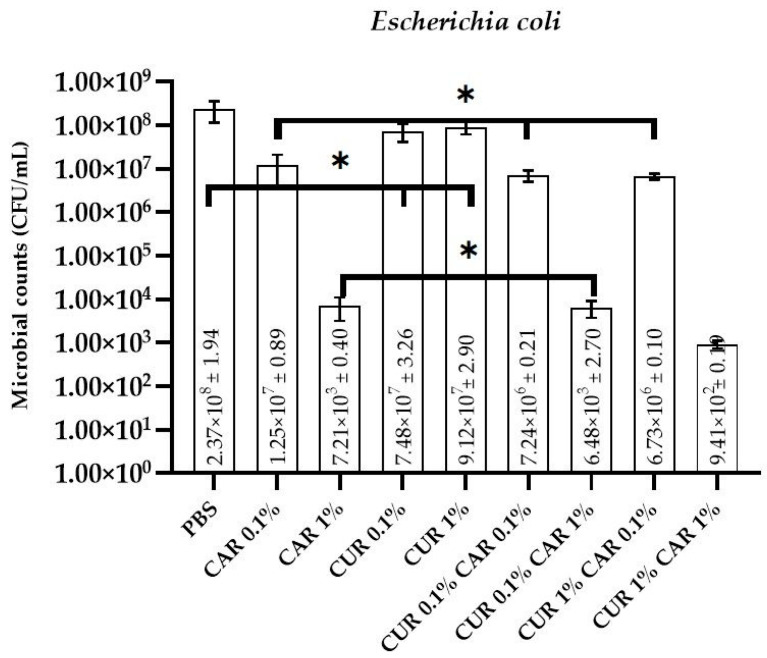
The effect of PBS-based neat and modified films on the viability of *Escherichia coli* cells; one-way ANOVA: *—*p* > 0.05.

**Figure 2:**
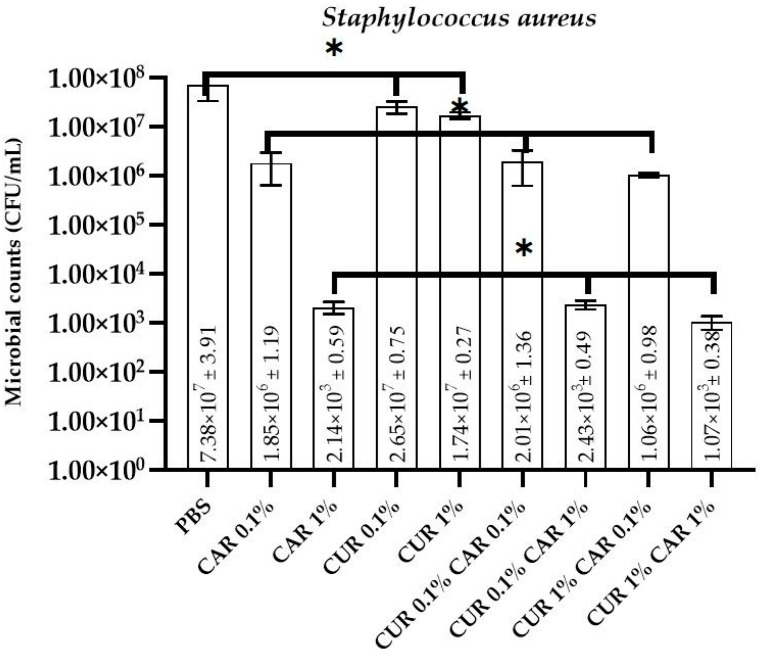
The effect of PBS-based neat and modified films on the viability of *Staphylococcus aureus* cells; one-way ANOVA: *—*p* > 0.05.

**Figure 3:**
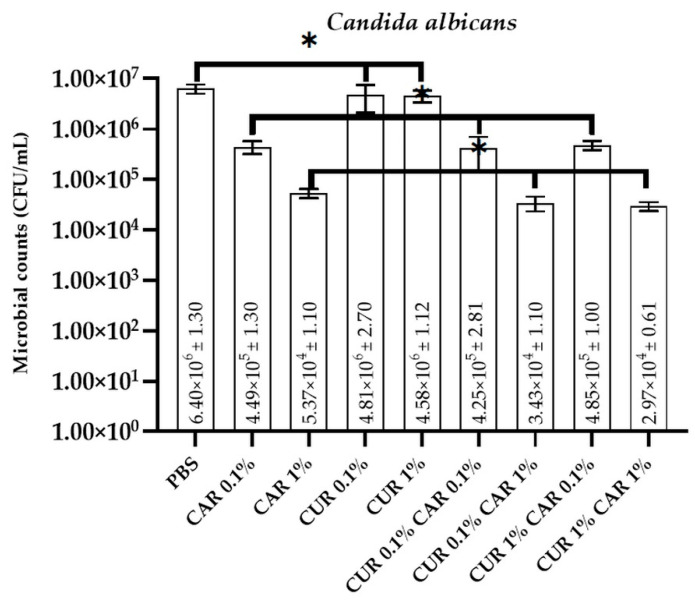
The effect of PBS-based neat and modified films on the viability of *Candida albicans* cells; one-way ANOVA: *—*p* > 0.05.

The antimicrobial activity of neat and modified PBS films towards bacteria (*Escherichia coli* and *Staphylococcus aureus*), as well as fungi (*Candida albicans*), is presented in Figure 1, Figure 2 and Figure 3. As expected, the neat PBS film did not exhibit any antibacterial and antifungal activity (2.37 × 10^8^ ± 1.94 CFU/mL, 7.38 × 10^7^ ± 1.91 CFU/mL, and 6.40 × 10^6^ ± 0.13 CFU/mL for *E. coli*, *S. aureus*, and *C. albicans*, respectively). For *S. aureus* and *C. albicans*, no antimicrobial effect was observed for both variants modified with curcumin (0.1% and 1%) (*p* > 0.05), whereas for *E. coli*, a very weak reduction of microbial counts was noticed (*p* < 0.05). Although CUR is known to have antimicrobial activity, its hydrophobic nature and insolubility in water limit its efficiency [46]. This observation is generally in line with the findings of Musso et al. [42], who observed that gelatin/curcumin films did not exhibit antimicrobial activity against *E. coli*, *S. aureus*, *S. enteritidis*, and *Bacillus cereus*. The lack of antimicrobial activity of gelatin/curcumin films was partly due to the low concentration of curcumin (0.4 wt% relative to gelatin), and also due to the interaction between curcumin and gelatin. Similarly, Barros et al. reported that free curcumin did not have any antibacterial effect on *Pseudomonas putida* [44]. Qiao et al. reported antimicrobial activity of PVB films when a high concentration (5%) of CUR was used [47]. In order to improve the solubility of CUR and enhance its antimicrobial mode, several delivery systems have been reported, such as the application of nanocarriers, inclusion complexes, liposomes, solid lipid nanoparticles, microemulsions, ionic liquids, and dimethylsulfoxide [12,44,46]. In fact, Roy and Rhim [27] reported outstanding antimicrobial activity of gelatin/curcumin composite films even at a low concentration of curcumin (0.25 wt%). The difference in antimicrobial activity of the gelatin/curcumin films probably resulted from the difference in the distribution of curcumin in the film matrix. In their study, treatment with the sodium dodecylsulphate (SDS) emulsifier seemed to increase antimicrobial activity by dispersing curcumin more uniformly in the polymer matrix, resulting in increased contact with the test bacteria. However, due to the fat solubility of curcumin, the possibility of its migration into fat-containing products, such as dairy products (e.g., cheese), and thus, possible interactions with the microbiota naturally present on these products, but also with food-borne pathogens, should be taken into account. Due to possible migration from materials (which requires further in-depth research), it is also potentially possible to deliver increased amounts of bioactive components with food, which may affect the microbiota of consumers as curcumin is active on the human microbiota [48]. For example, a recent study revealed that the *Curcuma longa* extract with a high curcumin content modulates the microbiota of patients with hypertension by improving the ratio of SCFAs, which strongly influences the *Enterobacteriaceae* group and negatively influences the *Bacteroides*-*Prevotella*-*Porphyromonas* groups [49].

On the contrary, a significant reduction (*p* < 0.05) in the number of viable cells was observed for PBS/CAR films (7.21 × 10^3^ ± 0.41 CFU/mL, 2.14 × 10^3^ ± 0.59 CFU/mL, and 5.37 × 10^4^ ± 0.11 CFU/mL, for *E. coli*, *S. aureus*, and *C. albicans*, respectively). CAR is well known for its great antimicrobial properties due to interactions with the cytoplasmic membranes of bacterial lipids and disruptions in fungal membranes [18,31]. It can be concluded that CAR has an antibacterial and antifungal effect in the PBS polymer matrix. These properties have been previously documented in the literature for films prepared from CMC [22], starch [15], or polypropylene [21]. There are no significant differences in microbial inhibition for CUR0.1%CAR0.1%, CUR0.1%CAR 1%, or CUR 1%CAR0.1% films compared to analogous films containing only CAR (*p* > 0.05). Only for *E. coli* for CUR 1%CAR 1% film, an increased inhibition of microorganisms was noticeable (from 7.21 × 10^3^ ± 0.41 CFU/mL for CAR 1% to 9.41 × 10^2^ ± 0.19 CFU/mL for CUR1%CAR 1%; *p* < 0.05), whereas for *S. aureus* and *C. albicans*, no such dependence could be observed (*p* > 0.05). It can be assumed that, in the case of *E. coli*, a synergistic effect of CUR and CAR in 1% concentration occurred, which improved the antimicrobial properties of the film; however, this mechanism requires further in-depth studies.

Biofilm formation is a problem in many areas of daily life. Wounds infected by biofilm-forming microorganisms are extremely difficult to treat (antimicrobial treatments are usually directed against planktonic cells, whereas biofilm can be thousands of times more resistant to treatment) and often lead to the need for amputation of infected limbs [50]. In addition, biofilm-forming microorganisms pose a threat to the food industry by infecting food and all kinds of equipment used in food processing [51], or by settling on the surface of water pipes, leading to contamination of drinking water [52]; hence, any way to combat biofilms is gaining the attention of researchers and industry.

**Figure 4:**
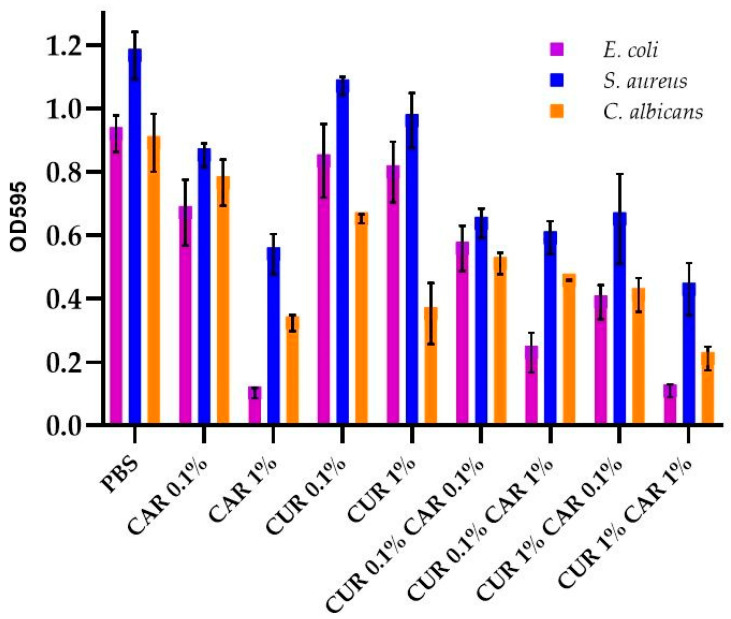
The effect of PBS-based neat and modified films on biofilm formation.

Figure 4 shows the effect of neat PBS film and modified films on single-species microbial biofilm formation. The addition of CAR to the PBS films resulted in a significant reduction in biofilm formation on the surface of the material (*p* < 0.05), which agrees with literature reports on the effect of CAR on the viability and formation of biofilms, including multispecies microbial communities [18]. Further, the addition of CUR resulted in a decrease in biofilm to a lesser extent than CAR, but the decrease was still statistically significant (*p* < 0.05). CUR has been shown to affect biofilm formation by affecting quorum-sensing when it is released from the polymer matrix [44,53]. The synergistic effect of CUR and CAR additives on biofilm formation is clearly dependent on the concentration of additives used for each of the three microorganism strains used. Films containing CUR 1%CAR1% showed the best inhibition of biofilm formation on the surface of the films, which gives reason to suspect that these additives act synergistically to complement each other, and to obstruct the adhesion of microorganisms to the surface of the material or communication between microbial cells.

### 3.2. Radicals Scavenging Activities and Reducing Power

The antioxidant activity of the films, quantified as the reducing power, as well as the radicals scavenging activity of DPPH and ABTS, are summarized in Table 2. The control PBS film showed no reducing ability or free radicals scavenging activity, as it was previously shown [43]. The addition of carvacrol significantly enhanced reducing power (*p* < 0.05), as well as both DPPH and ABTS radicals scavenging (*p* < 0.05). Similar radicals scavenging properties were demonstrated for CMC [22], PVA [54], PLA [13], and gelatin [41] films modified with carvacrol. Curcumin had a significant effect on all three parameters studied, significantly improving the antioxidant properties of the films, which was also shown in the literature for materials such as gelatin [27], PBAT [28], PLA [29], and tara gum/polyvinyl alcohol [12]. The excellent antioxidant function of curcumin is due to the donation of the H atom from the phenolic group. Similarly, Musso et al. [42], as well as Roy and Rhim [27], also reported that the addition of curcumin increased the antioxidant properties of gelatin-based films. In addition, a dose-dependent increase in antioxidant activity was observed for CUR and CAR, which is in line with previously reported findings [22]. It is worth noting that the combination of CUR and CAR showed synergistic mechanisms and significantly improved the antioxidant properties of the films (*p* < 0.05). They showed better results than for films with the same concentrations, but with only one active ingredient used, indicating a synergistic effect of curcumin and carvacrol. This observation is in line with the findings of a previous study where two antioxidants (carvacrol and melanin) were used in CMC films [22]. This fact gives reason to conclude that it is possible to achieve satisfactory antioxidant properties of films based on polymeric matrices using the synergistic effect of two active components with lower concentrations of these components than would be the case if these components were used separately.

## 4. Conclusions

As a result of the study, the antimicrobial, antibiofilm, and antioxidant activity of PBS-based films modified with carvacrol and curcumin, alone or in combinations, was shown. Carvacrol showed higher influence on antimicrobial activity, whereas curcumin showed higher influence on the antioxidant properties of the films. Antimicrobial efficacy synergy was observed against *E. coli*. The significant synergistic effect of the two active ingredients at lower concentrations than would occur if the ingredients were used separately may be of particular importance from an economic standpoint. In conclusion, multifunctional materials were obtained by using both compounds together. At present, further in-depth studies should be carried out before addressing new uses (especially to determine the suitability of the materials in food packaging, including migration tests into food stimulants and food products) and to determine the influence of bioactive compounds on heat-sealing strength and mechanical, thermal, and optical properties, as well as oxygen and water vapor permeability. Moreover, as this study demonstrated the preparation of films in a laboratory scale from a film-forming solution based on chloroform, further tests to obtain modified films by other methods (e.g., machine cast film extrusion method) without harmful solvents should be carried out to determine the possibility of large scale production of the films.

## Figures and Tables

**Table 1:** CUR and CAR concentrations (***w*/*w***) per weight of PBS used.

Sample Name	CUR Concentration (*w*/*w*)	CAR Concentration (*w*/*w*)
PBS	0%	0%
CAR1%	0%	1%
CAR0.1%	0%	0.1%
CUR0.1%	0.1%	0%
CUR1%	1%	0%
CUR0.1%CAR0.1%	0.1%	0.1%
CUR0.1%CAR 1%	0.1%	1%
CUR1%CAR0.1%	1%	0.1%
CUR1%CAR 1%	1%	1%

**Table 2:** Reducing power (RP) and radicals scavenging activity of PBS-based films.

Sample	RP (700 nm)	DPPH (%)	ABTS (%)
PBS	0.000 ± 0.000 ^f^	0.00 ± 0.00 ^e^	0.00 ± 0.00 ^f^
CAR1%	0.169 ± 0.004 ^de^	47.90 ± 3.68 ^b^	88.84 ± 1.89 ^b^
CAR0.1%	0.169 ± 0.001 ^de^	20.38 ± 2.78 ^c^	28.72 ± 1.89 ^c^
CUR0.1%	0.173 ± 0.001 ^cd^	25.14 ± 3.76 ^c^	51.93 ± 4.00 ^d^
CUR1%	0.190 ± 0.001 ^b^	88.43 ± 2.74 ^a^	98.21 ± 0.42 ^a^
CUR0.1%CAR0.1%	0.175 ± 0.002 ^c^	52.99 ± 4.78 ^b^	76.93 ± 0.63 ^e^
CUR0.1%CAR 1%	0.176 ± 0.002 ^c^	68.22 ± 1.25 ^d^	91.07 ± 0.00 ^b^
CUR1%CAR0.1%	0.193 ± 0.004 ^ab^	89.31 ± 0.23 ^a^	97.77 ± 0.21 ^a^
CUR1%CAR 1%	0.196 ± 0.007 ^a^	91.47 ± 0.00 ^a^	99.21 ± 0.00 ^a^

Values are means ± standard deviation of triplicate determinations. Means with different letters in the same column are significantly different at *p* < 0.05.

## Data Availability

The data presented in this study are available on request from the corresponding author.
